# Collaborated effort against SARS‐CoV‐2 outbreak in China

**DOI:** 10.1002/ctm2.7

**Published:** 2020-03-31

**Authors:** Qiwen Yang, Yang Zhou, Jingwen Ai, Jinmin Ma, Fenglin Cao, Weifan Cao, Wengeng Zhang, Shifu Wang, Weijun Chen, Xiaoling Ma, Wenhong Zhang, Weimin Li

**Affiliations:** ^1^ Department of Clinical Laboratory Peking Union Medical College Hospital Chinese Academy of Medical Sciences Beijing China; ^2^ BGI PathoGenesis Pharmaceutical Technology Co., Ltd BGI‐Shenzhen Shenzhen China; ^3^ Department of Infectious Disease Huashan Hospital of Fudan University Shanghai China; ^4^ BGI‐Shenzhen Shenzhen China; ^5^ The Centre Laboratory of Hematological and Oncological Department, The First Affiliated Hospital of Harbin Medical University Harbin China; ^6^ School of Biomedical Sciences The Chinese University of Hong Kong Hong Kong; ^7^ Precision Medicine Center & Precision Medicine Key Laboratory of Sichuan Province West China Hospital Sichuan University Chengdu China; ^8^ Department of Children's Medical Laboratory Diagnosis Center Qilu Children's Hospital of Shandong University Jinan China; ^9^ Division of Life Sciences and Medicine The First Affiliated Hospital of USTC University of Science and Technology of China Hefei Anhui China; ^10^ Department of Respiratory and Critical Care Medicine West China Hospital Sichuan University Chengdu China

**Keywords:** COVID‐19, SARS‐CoV‐2

## Abstract

A previously unknown beta coronavirus, SARS‐CoV‐2, was discovered from a cluster of patients with pneumonia of unknown cause in Wuhan since the end of 2019. Ever since the start of COVID‐19, government administrations, academic institutions, and technology enterprises are under unprecedented cooperation in controlling this outbreak from pathogen identification, epidemic situation assessment, to outbreak containment. Timely identification, isolation, and whole‐genome sequencing of SARS‐CoV‐2 have laid the foundation for effective control of this novel infection. With the increasing case numbers worldwide, more real‐time information is emerging, changing our understandings to SARS‐CoV‐2 outbreak, and nonetheless refining the outbreak control responses. The efficient management of COVID‐19 requires global collaboration and an efficient share of information.

At the end of 2019, a previously unknown beta coronavirus was discovered from a cluster of patients with pneumonia of unknown cause in Wuhan, China. Until March 2, 2020, this newly discovered coronavirus, named SARS‐CoV‐2 by WHO on February 11, 2020 (formerly named as 2019‐nCoV), has infected over 80 000 people with over 10 000 severe COVID‐19 cases nationwide in China, and over 9,000 cases out of China. Government administrations, academic institutions, and technology enterprises are under an unprecedented cooperation in controlling this outbreak of novel infection, from pathogen identification, epidemic situation assessment, to outbreak containment. More than 30 000 medical workers nationwide in China have arrived at Wuhan, where the first case of infection was reported and the center of this outbreak in China, and the surrounding area in Hubei Province. Although the number of confirmed and suspected SARS‐CoV‐2 infected patients is increasing, timely identification, isolation, and whole‐genome sequencing of SARS‐CoV‐2 have laid the foundation for effective control of this novel infection without a doubt. Up till now, National Health Commission of the People's Republic of China has updated the treatment guideline to its sixth version.

## 1 NEW TECHNOLOGY PROMOTES QUICKLY RESPONSE TO THE DISEASE CONTROL

Readily accessible information on an outbreak at the beginning is of importance to understand the risk before the implementation of infection management and outbreak containment activities. Besides reports from the initial outbreak site, a summary of symptoms at the prodromal phase and epidemic data, information on the causing pathogen, especially the genetic information, build the foundation for the development of diagnosis methods, selection of effective treatment, and further monitoring of the outbreak. In retrospect of the previous outbreaks of the novel or rare virus in the 21st century, it usually took weeks to months for pathogen detection: SARS‐CoV and MERS‐CoV were discovered after 5 months of the first case; the genome of Western African Ebola virus from the “patient zero” was sequenced 2 months after the outbreak.

For SARS‐CoV‐2 outbreak, the Chinese Center for Disease Control and Prevention (China CDC) published six genome assemblies on Global Initiative on Sharing All Influenza Data on January 11, 2020, within 2 weeks after the first report. In the article later published in *New England Journal of Medicine*, China Novel Coronavirus Investigating and Research Team, led by the China CDC together with multiple hospitals, published virus isolation procedures, phylogenetic analysis results, and virion structure obtained by transmission electron microscopy.^1^ As mentioned in this article, the rapid identification of this novel virus and acquisition of the genome, which used to be hurdle for efficient infection management against rare or newly emerged pathogens, was significantly contributed to by the unbiased metagenomics next‐generation sequencing (mNGS) technologies, which have been adopted in pathogen detection and identification under clinical practice in recent years.

The disclosure of the virus genome assemblies by China CDC was quickly followed up by the involvement of genetic research institutions in genetic analysis and diagnosis method development. Within days, multiple research institutes and technology companies launched rapid diagnosis kits based on the published SARS‐CoV‐2 genome. The Chinese genomic tech giant, BGI Genomics, immediately launched a real‐time PCR diagnosis kit and published another four SARS‐CoV‐2 genome assemblies on China National GeneBank (CNP0000881), which were sequenced on BGI's own sequencing platforms DNBSEQ‐T7 directly from clinical samples, without isolation or enrichment of virus. Up till February 25, 2020, six real‐time PCR rapid diagnosis kits, one metagenomic next‐generation (mNGS) analysis system, and two IgM/IgG Joint Detection Kit (colloidal gold) has gone through emergency certification by the Chinese National Medical Products Administration for virus detection and infection confirmation.

Although the availability of the SARS‐CoV‐2 genetic sequence is the very first step to understand and control this outbreak, the quick response from the disease control administrations and the adaptation of new sequencing technology is undoubtedly an exemplified practice in combating an outbreak of novel pathogens at present, even in the future.

## 2 THE WIDELY COLLABORATION ACROSS INSTITUTES

In the traditional epidemiologic triad model, the transmission of infectious diseases occurs when the pathogenic agent leaves its previous reservoir through a portal of exit, is conveyed by a certain mode of transmission, and enters through an appropriate portal to infect a susceptible population. The availability of SARS‐CoV‐2 genetic sequence sheds light on the epidemic tracking in the mass population, which is the necessary prerequisite for the academic and medical community in thoroughly studying this outbreak, including the natural reservoir of novel virus, mode of transmission, incubation period, and susceptible population.

Two articles published in *The Lancet* provide some important information at the early stage of the outbreak. Jasper Fuk‐Woo Chan and colleagues studied a family cluster of six infected people who returned to Shenzhen, Guangdong province after traveling to Wuhan, confirming the mode of person‐to‐person transmission.^2^ This mode of transmission was theoretically supported by Xintian Xu and colleagues in a structural modeling analysis of the S‐protein SARS‐CoV‐2, confirming its strong binding affinity.^3^ The study by Chaolin Huang and colleagues, which included 41 people confirmed with SARS‐CoV‐2 infection and admitted to hospital in Wuhan, summarized clinical manifestations, laboratory and radiological information, age, gender, and provided early estimates of incubation periods.^4^ The newly published article by China Medical Treatment Expert Group for COVID‐19 enrolled 1099 cases with an important update on clinical and epidemiological information, emphasizing the difference of COVID‐19 toward SARS and MERS.^5^ Clinical descriptive studies and epidemiological research are now covering the characteristic of COVID‐19 from image manifestation, syndromes, and pathogenesis to transmission dynamics.^6^, ^7^, ^8^, ^9^ All these articles went through rapid peer review and published online, providing first‐hand peer‐reviewed information to refine the outbreak control activity and further risk assessment.

With the increasing case numbers, more real‐time information is emerging, changing our understandings to SARS‐CoV‐2 outbreak, and nonetheless refining the outbreak control responses. Genome analysis of SARS‐CoV‐2 demonstrated a closer relationship to a bat coronavirus, indicating possible zoonotic transmission.^10^, ^11^, ^12^ The exposure history to the Huanan Seafood Wholesale Market used to be an important epidemic clue at the early stage, yet the importance has decreased and even challenged, due to not only the occurrence of secondary infection cases but also the retrospective analysis of the first 41 cases. A short discussion published in *Science* challenged the hypothesis that Wuhan seafood market being the source of the novel virus spreading globally, based on the fact that 13 out of the earliest 41 reported cases had no link to the marketplace, suspecting the possibility of other origins of this virus.^13^ While the pathogen identification and the start of outbreak quarantine activities happened within weeks, the relocation of virus origin(s) and early transmission pathway may take a long time, calling for continuous collaboration of different institutions.

## 3 CHALLENGE AND OPPORTUNITIES

On February 28, 2020, WHO has raised the risk level of SARS‐CoV‐2 to very high. As the understanding of the virus grows deeper every day, both challenge and opportunity exist in the combat against SARS‐CoV‐2. The large medical demand and rapid diagnosis are under urgent needs not just in China but worldwide. Since currently approved PCR and mNGS diagnosis kits/systems require the involvement of technicians and take a relatively long time, the combined application of serology and nucleic acid test might be adopted for the rapid diagnosis of COVID‐19. Refinement of the current diagnosis methods, as well as the development of other techniques, is still in demand for timely SARS‐CoV‐2 detection.

Although the latest report of the WHO‐China Joint Mission confirmed 99.9% homology among 104 viral genomes in different localities, without a significant mutation,^14^ the possibility of virus mutation and presence of “super transmitter” still poses threat to the population worldwide. As an RNA virus, SARS‐CoV‐2 has the inherent feature of a high mutation rate, which provides the possibility for this newly discovered zoonotic virus to adopted high infectiousness and virulence. Close monitoring of the change in virus genome, as well as the outbreak containment campaigns, is still of great importance not only in China but also in other parts of the world. As the appointed third‐party institution for virus detection and mutation monitoring, BGI Genomics has deployed several sets of its newly launched ultra‐high‐throughput sequencing platform, DNBSEQ‐T7, in the genomic sequencing of SARS‐CoV‐2. Nevertheless, it still requires global collaboration and efficient share of information in the risk assessment.

The ongoing SARS‐CoV‐2 outbreak has undoubtedly brought back the memories and terror of the SARS‐CoV outbreak starting 17 years ago. However, the knowledge accumulated and technologies developed since then have been proved to be a new armor to human beings. Following the early studies published, virus genome from multiple countries has been studied, several China CDC regional branches have successfully isolated the virus with a high titer, and clinical trials of treatments and vaccines are undergoing, making important steps in understanding this virus and managing this outbreak.
